# School-Based Restorative Justice for Student Conflicts in China: Theoretical Logic and Local Adaptation

**DOI:** 10.3390/bs16081346

**Published:** 2026-08-05

**Authors:** Xuanyu Chen, Feng Han

**Affiliations:** 1 Law School, Beijing Normal University, Room 1805B, Haidian District, Beijing 100875, China; xychen@bnu.edu.cn; 2 Institute of Higher Education, Beijing University of Technology, 100 Pingleyuan, Chaoyang District, Beijing 100124, China

**Keywords:** restorative justice, restorative practice, student conflict, coexistence, relational justice

## Abstract

Conflicts are an everyday reality in the school environment. Student conflicts can escalate into bullying, violence, and injury disputes when schools rely solely on incident-focused or punitive responses. This conceptual, normative article examines how restorative justice might complement, rather than replace, disciplinary accountability in Chinese schools. Drawing on relational justice, behavioural science accounts of student conflict, international school-based restorative practice, and Chinese legal and policy materials, it advances three propositions. First, a relational account locates conflict in disrupted interpersonal and institutional relationships and directs attention to harm, needs, responsibility, and repair. Student conflict intervention should focus on more educational and relationship-restorative response pathways. Second, international evidence suggests that restorative approaches may support social–emotional learning, conflict-management skills, and school relationships; however, outcomes vary with programme quality, institutional support, school culture, and cultural context. Third, while the introduction of restorative justice into Chinese campuses appears to enjoy a degree of foundational compatibility with traditional cultural resources and the structural characteristics of the school environment, it also confronts challenges of conceptual adaptation and institutional integration that warrant careful scholarly attention. The article therefore proposes a layered model combining universal preventive education and proportionate restorative interventions. Restorative justice should be treated as a cautiously adapted supplementary framework whose feasibility and effects in Chinese schools require local empirical evaluation.

## 1. Introduction

Student conflict refers to interpersonal disagreement, opposition, retaliation, or resistance among individuals of similar age and is common in young people’s social interactions (D. W. Chen, 2003). It may involve physical conflict, verbal abuse (insults and slander), or, in some circumstances, school bullying (Johnson & Johnson, 1996). Students may exhibit aggressive behaviours when their needs for autonomy, belonging, or competence are frustrated, or when they have learned to resolve problems through violence. Such incidents are not isolated; they may reflect a mismatch between developing social needs and still-maturing capacities for behavioural and emotional regulation.

Concepts closely related to student conflict include school violence, school bullying, and student injury disputes. While these four concepts are interrelated, they remain distinct from one another. In school settings, violence refers to the use of force against any member of the educational community that results in, or has a high likelihood of resulting in, injury, death, psychological harm, developmental impairment, or deprivation (Pérez-Jorge et al., 2023). Violence and conflict are related, but not every conflict becomes violent (Gómez Nashiki, 2014). When conflicts are not addressed promptly and appropriately, they may escalate into more severe forms of aggression, with harmful consequences for children and adolescents (Pérez-Jorge et al., 2023; Rodríguez et al., 2015). The White Paper on the Procuratorial Work for Minors (2024) issued by the Supreme People’s Procuratorate of the People’s Republic of China (2025), indicates that violent offences—including affray (gathering crowds to engage in affrays), picking quarrels and provoking trouble, and robbery—collectively account for approximately 25% of all juvenile criminal offences. When such violent acts occur among students, they may be regarded as severe violent student conflicts.

School bullying involves intentional and repeated physical, verbal, psychological, or relational aggression directed at a student who is in a less powerful position (Smith, 2014). In academic research and in Chinese educational and legal practice, bullying is commonly regarded as one type of student conflict while remaining distinct from conflict more generally (Villalba Cano, 2016; X. M. Zhang, 2025). Physical, verbal, and psychological aggression, as well as relational exclusion, can harm the entire educational community (Falla & Ortega-Ruiz, 2019). Alternative approaches have therefore been developed to promote values education and constructive conflict regulation as a means of reducing violence (Villalba Cano, 2016). Some student conflict intervention programmes also serve as school-bullying intervention programmes (Pérez-Jorge et al., 2020).

Meanwhile, where conflicts lead to tangible harm, student injury disputes may subsequently emerge. Student injury disputes may be defined as civil controversies arising from physical or psychological harm suffered by a student during educational or teaching activities conducted by a school, kindergarten, or other educational institution, or during on- or off-campus activities organised or administered by such an institution, or within premises over which the institution exercises a duty of care. They may arise among students, between a student and the institution, or between a student and staff, and usually concern the facts of harm, causation, responsibility, and remedies. At present, student injury disputes triggered by student conflicts, as well as incidents of school bullying and school violence, have drawn growing attention in China (X. M. Zhang, 2025). In some cases, the mishandling of individual student conflicts may escalate into vicious juvenile criminal incidents that have drawn extensive social attention (S. G. Zhang, 2024). For example, in the Handan case, following a series of everyday conflicts, three middle-school students inflicted severe facial injuries on a classmate and subsequently killed the victim and buried the body in an abandoned vegetable greenhouse (Hebei Handan Reports, 2024). After the incident, related posts on the social media platform Sina Weibo reportedly received 260 million views and generated 79,000 discussions (Youxun Public Opinion, 2024).

It follows that effective intervention in, and resolution of, student conflicts play a vital role in preventing school violence and bullying and mitigating student injury disputes. Providing appropriate responses to student conflicts and preventing their escalation are important issues in educational rule of law and the protection of minors.

Moreover, student conflict itself is neither positive nor negative and can be constructive. In the context of school coexistence, systematic reviews suggest that conflicts should be seen as opportunities for change (Villalba Cano, 2016). Several scholars point out that conflict can serve as a driving force of students’ cognitive and moral development (D. W. Chen et al., 2001). Many young people’s attacks on their peers are closely related to a lack of skills and strategies for adequately solving problems (Rueda Carcelén et al., 2016). In 2014, in the Education for All Global Monitoring Report, UNESCO stated that conflict resolution skills are essential transferable competencies for students as global citizens (UNESCO, 2014). Schools are the most relevant socialisation scenarios for the growth of primary and secondary school students (Pérez-Jorge et al., 2022). By embedding shared values into the school culture, educators can cultivate an equitable and respectful climate conducive to constructive interaction, nurture a broad spectrum of competencies essential for individual growth, and advance the holistic development of every student.

Traditionally, discipline has been understood as adherence to school or classroom rules intended to maintain the order necessary for teaching and learning (Morrison et al., 2005). Schools prevent and handle conflicts by formulating and implementing disciplinary measures, and the law also attempts to discipline unruly students by strengthening educational punishment. However, punitive and disciplinary measures are only one approach to problem solving. The goal of student conflict resolution is to resolve conflict and restore relationships rather than merely punish bad behaviour (Avivar-Cáceres et al., 2022). On this basis, over the past two decades, a growing number of countries have adopted programmes and practices that are centred on the concept of restorative justice to prevent and handle student conflicts, thereby contributing to the improvement of coexistence and the strengthening of affective bonds between the individuals involved in such situations (Esquivel Marín, 2018), which facilitates the positive resolution of conflicts. Studies from multiple countries report potential benefits of restorative approaches; however, the strength and consistency of reported effects vary across study designs, implementation conditions, and school contexts (McCluskey, 2010; Alonso-Rodríguez et al., 2025).

Restorative justice was introduced into China in 2002 (Z. X. Wu, 2002). Subsequent research on restorative justice has focused mostly on the handling of environmental crime, juvenile delinquency, and the execution of criminal penalties (Z. X. Wu, 2023), and few applications in the field of education have been reported aside from the prevention and control of school bullying. Restorative justice is a form of justice grounded in “social–emotional intelligence” (Sherman, 2003) aimed at resolving conflicts through dialogue and interaction among the parties to protect victims and promote social reconciliation. Its core objectives are to hold offenders accountable, repair harm, commit to behavioural change and repair relationships, and it serves as a complementary approach in which peaceful strategies are adopted to resolve conflicts. The values of “healing” and “restoration” advocated by restorative justice not only align with the supreme social ideals of “no litigation” and “harmony” pursued in traditional Chinese legal culture and are compatible with the construction of “emotional communities” in schools, but also conform to the orientation of China’s current educational policies.

In terms of student conflict resolution, the 2022 National Guidelines for Creating Model Schools Governed by Law (Primary and Secondary Schools) stipulate that for decisions affecting students’ vital interests, representatives of relevant stakeholders should be invited to attend or participate in decision-making meetings. The rule of law should serve as the basic means of resolving campus conflicts, with mediation and other approaches used comprehensively to address issues lawfully, appropriately, and efficiently. The General Office of the Ministry of Education of the People’s Republic of China (2025) calls for the timely resolution of conflicts among students in its Ten Measures for Further Strengthening Mental Health Work for Primary and Secondary School Students (Document No. 教基厅〔2025〕2号). In terms of resolving student disputes stemming from conflicts, the 15th Five-Year Plan for Education Development reiterates the commitment to improving the diversified dispute resolution mechanism for campus safety disputes, guiding parents to handle student disputes in a rational manner, and advancing the resolution of disputes at the front end while addressing them at the source. Similarly, the Standards for the Management of Compulsory Education Schools require the establishment of consultation mechanisms to gather opinions and suggestions from students, staff, and parents to effectively address conflicts in terms of conflict prevention education. The 2024–2035 Master Plan on Building China into a Leading Country in Education reaffirms the in-depth implementation of quality-oriented education (Huai, 2025). Laws related to minors (e.g., Article 25 of the Law on the Protection of Minors, Article 3 of the Compulsory Education Law) also emphasise that schools should focus on cultivating minor students’ cognitive and cooperative abilities to advance their holistic development.

Restorative justice is inherently aligned with the above-mentioned policy orientation towards the prevention and resolution of student conflicts. The principle of inclusive participation inherent in restorative justice requires that all stakeholders be brought into the dialogue process, a feature that is fully consistent with the requirements of school governance under the rule of law that stakeholders participate in decision-making and that parents be involved in dispute resolution. Its relationship-oriented and forward-looking orientation treats conflict as an educational opportunity, repairing relationships, correcting cognitive distortions, and preventing the escalation of conflict through dialogue, thereby aligning with the policy objectives of front-end resolution and source-based disposition of disputes. The educative character of the process itself transforms conflict intervention into a practical field for cultivating students’ empathic capacity, communication and cooperation skills, and civic literacy, directly responding to the requirements of quality-oriented education and the development of cognitive-cooperative competence. Moreover, as a dialogue mechanism situated at the base of the regulatory pyramid under responsive regulation, restorative justice prioritises negotiation and cooperation, thereby enriching the toolbox for diversified resolution of campus safety disputes. In sum, restorative justice integrates fragmented policy requirements into a coherent framework centred on “relationship repair,” shifting student conflict intervention from individual blaming to collaborative restoration and from ex post disposition to relational regulation, thereby furnishing systematic methodological support for the construction of Safe Campus and the rule of law in education.

In view of the continuing interest in restorative justice in legal and educational scholarship and the need for more developmentally responsive responses to student conflict, this article examines whether restorative justice may complement existing approaches in Chinese schools. The article develops its argument through four analytical stages. First, it establishes the conceptual foundations of school-based restorative justice, providing the normative basis for extending restorative justice from criminal justice to student conflict intervention in schools. Second, the article develops a theoretical framework for student conflict intervention. It connects self-determination and social learning theories to restorative intervention and reviews international school practices to identify reported outcomes, possible behavioural mechanisms, and implementation conditions. These international studies inform practice in Chinese schools. Third, the article conducts an argumentative synthesis of local adaptation in Chinese schools. It examines the limitations of current student conflict intervention and considers how traditional cultural resources, school organisational conditions, and educational laws and policies interact. This analysis considers both opportunities and risks, including pressured reconciliation, unequal participation, and weak victim protection. Finally, it discusses the conditions for safeguarded implementation. Drawing on the preceding conceptual, behavioural, international, and local analyses, it sets out a staged model for Chinese schools.

## 2. Approach

This study adopts a normative legal analytical paradigm to examine whether, why, and under what institutional conditions restorative justice may be introduced into student conflict governance in Chinese schools. The analysis uses normative legal reasoning and contextual comparative analysis, supplemented by purposively selected theoretical, behavioural, international, and Chinese institutional materials.

The analysis draws on four bodies of material with distinct analytical purposes. First, foundational theoretical scholarship on restorative and relational justice, including the work of Llewellyn, Braithwaite, Zehr, and Morrison, establishes the normative basis for extending restorative justice from criminal justice to schools. Second, behavioural science theory and empirical research on need frustration, social learning, emotion regulation, and peer relations are used to explain psychological and social mechanisms through which student conflict may emerge, escalate, and change. Third, international systematic reviews, meta-analyses, intervention and evaluation studies, and practice-oriented accounts of school-based restorative work are used to identify reported outcomes, implementation conditions, and limitations. These sources provide comparative references for assessing practical feasibility. Fourth, Chinese laws, policy documents, published judgement analyses, and publicly available cases relevant to student conflict and school governance are used to assess institutional fit, constraints, safeguards, and local adaptability.

The literature was located through CNKI, Google Scholar, SAGE Journals, ProQuest, and Scopus. Sources were selected primarily on the basis of conceptual relevance, empirical relevance to school settings, and representativeness where possible. Chinese laws and policy documents were retrieved from official government portals, including the Law on the Protection of Minors, the Compulsory Education Law, the 15th Five-Year Plan for Education Development, and the 2024–2035 Master Plan on Building China into a Leading Country in Education. This article is not a systematic, scoping, or narrative review. It does not use a PRISMA flow process, risk-of-bias assessment, or pooled effect-size analysis; the materials are used solely for conceptual and normative analysis.

In addition to published literature and official texts, the study incorporates practice-based observations and illustrative case materials concerning school conflict intervention practices in China. These include observations derived from law popularisation activities attended by the authors and publicly documented civil cases. Their function is limited to providing contextual grounding and illustrative support for the normative and comparative analysis, thereby connecting the institutional discussion more closely with the realities of school governance in China.

The materials are interpreted through normative legal reasoning, contextual comparison, and inductive and deductive analysis. They are analysed sequentially to establish the conceptual foundations of restorative justice, explain behavioural mechanisms, identify implementation conditions, assess local adaptation in China, and derive safeguarded implementation propositions.

## 3. Conceptual Foundations: Restorative Justice and Relational Justice

Restorative justice has diverse meanings and is applied in a variety of contexts. Since Albert Eglash first coined the term “restorative justice” in 1977, more than four decades have elapsed, and restorative justice is no longer regarded merely as an established programme or blueprint but rather as a set of “people and programmes that employ restorative processes and seek to achieve restorative results” (Zhou & Wang, 2010), and it is applicable to criminal justice, education, families, and other fields.

### 3.1. The Concept of Restorative Justice

Restorative justice is more commonly translated as “恢复性司法” on the Chinese mainland, “复合公义” in Hong Kong, and “修复式正义” in Taiwan (Wang, 2016). In this paper, the term “修复” (restorative) is used in the school context. In addition to its implication of the idea of “striving to make things better” (X. Y. Chen & Han, 2025), the term is more fundamentally derived from the core of restorative justice, which lies in rebuilding relationships (Zehr, 2005). Wrongdoing is often not only a cause of inequality but also a product of existing relational dynamics. Thus, restoring a situation to a previous state, in most cases, would fail to address the problems in the relationship that enabled or perpetuated the initial conflict (Llewellyn & Howse, 1999). For example, bullying is essentially a relational problem of domination (Burford et al., 2019). The key to resolving such conflicts is to examine the relational state at the time of the wrongdoing and strive to reshape it into an ideal equal relationship. Furthermore, since “justice” has multiple meanings—fairness, righteousness, and judicial administration—it is consistent with academic practice and more accurate to use “修复正义” (restorative justice as an idea) at the conceptual level and “修复性司法” (restorative justice in the legal field) or “修复性实践” (restorative practices in the educational field) in practice. These distinctions are also maintained in the discussion section of this paper.

Restorative justice spans different fields and thus has many related definitions. In its early stages, it was defined as “a process whereby all the parties with a stake in a particular offence come together to resolve collectively how to deal with the aftermath of the offense and its implications for the future” (Marshall, 1996). In 2002, the United Nations Commission on Crime Prevention and Criminal Justice defined the term as “any program that uses restorative processes and seeks to achieve restorative results” (Wang, 2005), noting that member states could reach consensus on its core ideas rather than on its specific elements (Tu & Liu, 2025). In recent years, with the expansion of its application fields, restorative justice has come to refer to participatory approaches that bring relevant parties together in respectful dialogue to discuss what happened, who was affected and how, and what actions need to be taken to correct the harm done (Morrison, 2003). Restorative justice is focused on fundamental questions such as who was harmed, what the needs and obligations of all affected parties are, how to come to a consensus on ways to heal the harm and what the causes of conflicts were (Zehr, 2002). It is centrally concerned with understanding and addressing the harm resulting from a wrong (Llewellyn & Downie, 2011) and emphasises the importance of relationships (Morrison et al., 2005).

### 3.2. Theoretical Basis of Restorative Justice

Restorative justice is a relational theory of justice (Llewellyn, 2011). In applying this theory, relationships are to be treated as a factual reality that needs to be considered (Llewellyn, 2011) rather than as an independent value to be maintained or promoted. Relational theory indicates that there is some kind of connection between people, whether good or bad (Llewellyn, 2021). The justice of interaction patterns and institutional arrangements at the interpersonal, institutional, and systemic levels depends solely on how such relations are structured (Llewellyn & Morrison, 2018). Thus, the harm caused by wrongdoing is not merely a violation of individual rights but also possesses relational attributes. Its damage to interpersonal trust, community bonds and norms of coexistence is often more far-reaching than its visible behavioural consequences.

Thus, the goal of addressing harm is not simply to compensate for a specific “punch” but also to pay attention to human relationships and their structural forms, relying on the repair of interpersonal relationships to terminate certain harmful relations or patterns (Zhou & Wang, 2010). To achieve justice, it is necessary to simultaneously repair the specific harm, rebuild relational patterns, and restore social relations. The traditional disciplinary model simplifies complex conflicts into uniform rule applications through punishment; however, in response to the disposition of specific behaviours, it empties the situation of its relational context and cannot thoroughly resolve the problem. The core of addressing wrongdoing should be the establishment or reshaping of restorative relationships characterised by equal respect and mutual concern on the basis of the construction of relations in which all involved parties enjoy equal status (Llewellyn & Howse, 1999).

Within the framework of restorative justice, to fully clarify responsibilities and achieve meaningful accountability, it is necessary to present a comprehensive picture of the incident in a specific context. This includes all the details, such as the course of the event, the involved subjects, and the affected parties, to understand the relational context of the conflict (such as power imbalances and social exclusion). This not only requires restorative justice to focus on specific harmful incidents or interpersonal conflicts but also dictates that the handling procedures must fully take the background, causes, and specific circumstances of the incident into account to ensure comprehensive and integrated responses (Llewellyn, 2019). During this process, those who cause or contribute to harm have a responsibility to understand the impacts of their conduct and to participate actively in the development and implementation of solutions to repair the harm.

It should be emphasised that restorative justice does not abandon accountability; rather, through a strategic orientation that shifts the primary focus away from attributing blame, it achieves a more profound grasp of responsibility. The traditional punitive model labels the offender as a “rule violator”, excluding them from the community through condemnation and punishment and relegating them to a state of marginalisation. Although the penalty is executed, the offender is afforded no opportunity to repair the harm or rebuild broken bonds, leaving the underlying conflict unaddressed. In contrast, restorative justice transforms the determination of right and wrong into an opportunity for relationship repair:rather than being stigmatised, the offender confronts the consequences of his or her actions, listens to the victim’s account, comprehends the impact of the harm, and participates in the negotiation and implementation of a reparation plan, thereby gradually assuming responsibility to both the victim and the broader community. Throughout this process, the offender is regarded not as a member who should be excluded but as one who is capable of reform. On the basis of acknowledging wrongdoing and accepting responsibility, the offender is transformed from a person who caused harm into a person who actively participates in repair, is reintegrated into the community’s relational network, and is restored to the social order of coexistence.

Specifically, the practical features of restorative justice fall into four progressive dimensions (Llewellyn et al., 2013). The first dimension is a relationship-oriented holistic perspective that avoids viewing individuals or events in isolation but instead integrates backgrounds, causes, and environmental factors to understand the full picture of the event from the perspectives of all involved parties. The second dimension emphasises the inclusiveness, participation, and empowerment of agency, whereby all the subjects with a stake in the outcome are incorporated into the procedure; through dialogical processes, the voices of all the parties are heard, their needs are respected, and their agency is facilitated. The third dimension involves collaborative accountability, which fosters an atmosphere of equal communication and dialogue, emphasises the fulfilment of accountability rather than simply assigning blame, and encourages all parties to jointly identify reparative pathways through collaboration rather than confrontation. The fourth dimension encompasses responsiveness and a forward-looking orientation, which attends to specific contexts and the differentiated needs of all parties; it transcends backwards-looking punitive logic to emphasise educational guidance, problem prevention, and proactive intervention and thereby creates constructive possibilities oriented towards the future for all parties to the conflict.

These theoretical features suggest that restorative justice has the potential to contribute to the identification, prevention, and management of conflict across family, educational, and judicial contexts and promote more constructive coexistence (González Contreras et al., 2021).

## 4. Theoretical Framework: Behavioural Mechanisms and School-Based Restorative Practices

From a historical perspective, one field that fostered the emergence and development of restorative justice is the governance of problem youth and the juvenile justice system (S. H. Jiang & Zhang, 2024). Peace-oriented conflict resolution education and its related emphasis on relational justice as advocated by restorative justice align with schools’ educational and guiding obligations. The international literature suggests the value of restorative justice in addressing school problems and student conflicts.

### 4.1. Behavioural Science Explanations of Student Conflict

Restorative-practice scholarship has increasingly considered the application of restorative approaches in school settings (Bonell et al., 2014). In conceptual accounts of restorative practice, student conflict is understood as a problem of coexistence in which parties cannot reach a workable agreement and may respond aggressively. This conceptual claim is broadly consistent with behavioural science accounts of adolescent conflict; in this subsection, self-determination and social learning theories are used as explanatory frameworks.

According to self-determination theory (SDT), the more an individual’s confidence in his or her abilities and perception of effectiveness is satisfied during an activity, the stronger his or her autonomous motivation to participate becomes (Deci & Ryan, 2000). Conversely, when an individual’s needs for autonomy, competence, and relatedness are thwarted, they are more inclined to take measures to compensate for the frustration of these basic psychological needs and seek substitutes (Fang et al., 2022). For instance, when individuals fail to achieve a sense of competence in legitimate domains such as academics or social interactions, conflict behaviours, particularly physical altercations and bullying, can provide certain students with an experience of competence characterised by feelings that “I can control the situation” or “I am stronger than others.” When they are unable to achieve a sense of relatedness through prosocial avenues, they may participate in collective bullying to secure a sense of belonging, feeling that “we are in this together.” Moreover, when they perceive themselves as being treated unfairly, such as being wronged or marginalised, or having their interests compromised, this may trigger retaliatory conflict behaviours as a means to restore their sense of fairness.

Moreover, according to Bandura’s social learning theory, students’ conflict resolution behaviours are often acquired through observation (Bandura, 1977). During the process of learning and internalising behaviours, the social environment and interacting peers play a decisive role (Xu, 2026). If students are chronically exposed to environments where problems are resolved through violence or confrontation, they are more likely to internalise these patterns as their own behavioural habits. If the school environment is structured around the assumption that conflicts must be resolved through punishment, students’ expectations regarding behavioural consequences will lead them to suppress conflicts only in monitored situations (H. L. Zhang & Shi, 2017). However, this suppression is not self-endorsed; once the monitoring disappears, such as when a teacher turns away or leaves the classroom, the suppressed conflict behaviours will rebound in a more intense manner. Harsh punitive measures and exclusionary disciplinary tactics not only deprive students of valuable learning opportunities but also may exacerbate inequalities within the campus (González et al., 2019).

In addition, student conflicts are not isolated events. Schools create an interactive environment in which adolescents are required to follow norms shared with their peers, and peer interactions exert a diffusion effect (Guest & McRee, 2009). Every conflict that is not properly repaired undermines the coexistence norms of the school community. When other students observe that conflicts go unchecked, individual aggressive behaviours may not only be imitated but may also escalate from minor skirmishes into serious incidents such as bullying and violence. From the perspective of positive conflict cognition, parties in conflict share both disagreements and common ground rather than being in complete opposition; conflict resolution is not a zero-sum outcome in which one side must win and the other lose. Genuine peace can emerge through conflict resolution when parties pursue mutual gains through creative and constructive strategies.

Taken together, the theoretical and behavioural literature suggests that student conflict may emerge through the interaction of situational influences, communication skills, emotional regulation, and behavioural expectations. At the individual level, students must take responsibility for their own actions; they need to proactively recognise problems and properly resolve them. Knowledge reserves, situational coping abilities, and emotional and communication skills such as active listening and confident expression are key elements in effectively resolving conflicts (Benítez Moreno et al., 2024). At the environmental level, schools should cultivate positive interpersonal relationships and create a healthier, more cohesive campus environment (Bonell et al., 2015). It is necessary to examine whether the school system itself creates or tolerates unjust relational structures, for example, whether the disciplinary system relies excessively on exclusionary punishments, which causes students to merely restrain conflicts under surveillance instead of genuinely internalising norms of coexistence. These theories provide a rationale for examining individual behavioural change and school climate together. Theoretical work further proposes that schools can treat conflict as an educational opportunity for developing emotional, interpersonal, and non-violent communication skills (Alonso-Rodríguez et al., 2025). This article therefore argues that restorative approaches offer behaviourally plausible pathways for such work.

### 4.2. Theoretical Logic of Using Restorative Justice to Intervene in Student Conflicts

Relational, procedural-justice, and reintegrative-shaming scholarship is used to specify plausible mechanisms of restorative intervention. First, the aforementioned relational justice theory establishes the conceptual basis for restorative justice intervention in student conflicts. Student conflicts are endogenous to interpersonal relationships, and any harm in a conflict is not merely a violation of isolated individual rights but a tearing of the relational network itself. The pain caused by conflict stems not only from physical injury or property loss but also from the existential crisis brought about by relational rupture, such as the feeling of no longer being accepted, no longer being safe, or being exiled from the group. Therefore, the core of intervening in student conflicts is relational repair. Nelson Mandela’s adage, “I destroy my enemies when I make them my friends”, captures the profoundly inclusive nature of restorative justice (Davis, 2014). By bringing together subjects with seemingly opposing stances through well-designed face-to-face communication, all parties listen and express themselves with respect and sincerity. They discuss specific relationships in specific contexts, understand the relational threads of the conflict (such as background factors such as power imbalances, social exclusion, and observational learning), develop empathetic understanding, and ultimately reach a consensus on problem-solving. This restorative practice emphasises reparation for harm, accountability, and the reintegration of the individual (Zehr, 2015). It not only enriches the educational environment but also provides opportunities to promote inclusion, acceptance, and mutual respect (Pérez-Jorge et al., 2021), moving towards a more just relational structure or transformation.

Second, Tyler’s theory of procedural justice broadens the theoretical foundation for restorative justice from the perspective of institutional functioning (Tyler & Blader, 2000). Within Tyler’s framework, the core of procedural justice lies in enabling participants to feel respected and heard while exercising substantive voice in the decision-making process—an experience directly linked to individuals’ sense of identification with and pride in their group membership. People value justice because of their concern for social status. Students are extremely sensitive to fairness in interpersonal relationships. Young people need environments where they feel valued and accepted, which is a crucial part of their transition from childhood to adulthood (Morrison, 2002). By providing students with ample opportunities to express their feelings and thoughts, restorative justice establishes an inclusive and respectful dialogue framework and rebuilds both parties’ perceptions of fairness regarding the handling process through procedural justice. When students feel respected in restorative procedures, their sense of identity and belonging to the collective (e.g., the class) is strengthened. Restorative justice effectively responds to the demands for power and status during adolescent development and satisfies the needs for competence, relatedness, and autonomy. Subtly, the school climate and the coexistence skills of the students will also be simultaneously improved (Villalba Cano, 2016).

Finally, Braithwaite’s reintegrative shaming theory (Braithwaite, 2002) explains the effective operation of restorative justice in student conflict intervention from the perspective of psychological mechanisms. According to this theory, when participants release the shame triggered by misconduct in a respectful manner, they can more profoundly recognise the wrongfulness of their behaviour, establish positive self-identity, and prevent themselves from reoffending by improving their cognition, discernment, and self-control (Kong & Wang, 2024). It is difficult for the offending party to acknowledge the harm and the community relationship because it requires letting go of defensive self-narratives, such as claiming that the other party provoked them first or that it was unintentional, and facing the real impact of their behaviour on others. In the course of restorative dialogue, listening face-to-face to the victim’s statements can develop the wrongdoer’s empathetic understanding, triggering remorse, shame, and guilt (Harris et al., 2004). Through communication based on equality and respect, students are guided to understand the harm caused by their actions, assume the corresponding responsibilities, and actively make changes (Alonso-Rodríguez et al., 2025). Students’ sense of responsibility, social–emotional skills, autonomy, and tolerance naturally emerge through this understanding (Prutzman et al., 2022).

On the basis of the above theories, different variations among restorative methods share key principles such as fairness, respect, responsibility, inclusion, and reintegration (Zehr, 2015). Accordingly, programmes promoting restorative educational concepts emphasise visibility and the creation of a safe, compassionate, and inclusive environment, advancing towards a more equitable relational framework.

### 4.3. International Experience: Reported Outcomes and Implementation of Restorative Justice in Schools

Since the first school-based conference was held in a Queensland (Australia) school in 1994 (Morrison et al., 2005), the practices of restorative justice in schools have flourished in the USA, England, Wales, Australia, and Canada. Restorative practices in schools can be divided into two categories.

The first category consists of prevention- and education-oriented curricula and training programmes, including social–emotional learning programmes, those focused on values and respect, or those that address specific problems such as bullying (Pérez-Jorge et al., 2023). These programmes adopt educational methods that cultivate social–emotional skills such as empathy, self-esteem, nonviolent communication, and conflict management (Lodi et al., 2022), and they are aimed at improving students’ conflict management and dialogue skills, creating a happy and harmonious school atmosphere, and providing a platform that enables teachers and students to properly handle conflicts.

The second category covers restorative justice processes for responding to conflicts, misconduct, and violent behaviours, which include informal restorative practices and formal restorative justice processes. Typical examples of the former include peer mediation and both classroom and corridor conferences (Wachtel & McCold, 2001). In these mediation/peer help systems, students intervene from a position of neutrality, seeking a balance between the parties involved without imposing value judgments or preset solutions, and returning the right to speak to those involved. The latter includes restorative conferences involving victims, offenders, families, schools, and mediators, such as victim–offender dialogue, peacemaking circles, and victim impact panels. Through these processes, involved students can fully express their worries, pain, anger, hatred, shame, apologies, and demands for reparation (S. H. Jiang & Zhang, 2024), thereby seeking collaborative alternatives to solve conflicts (McCold & Wachtel, 2002), taking responsibility, and repairing and healing harm.

Studies in multiple jurisdictions have reported that restorative justice and practices are not only aimed at seeking solutions to conflict but also at helping to develop new strategies for managing social relationships and the classroom environment to promote students’ mental health and integral development (Mas-Expósito et al., 2022). Empirical observations by Morrison in schools implementing restorative practices indicate that restorative justice is an opportunity for all the involved parties to learn and grow together, thereby improving their resilience, responsibility, conflict handling, and accountability (Morrison, 2003). An intervention study conducted by Pérez-Jorge et al. involving 336 students in Mexico indicates that after students participated in a dispute prevention project using the restorative approach, they showed improvements in emotional self-regulation, self-determination and the use of peaceful strategies to resolve conflicts among students (Pérez-Jorge et al., 2023). A meta-analysis conducted by Durlak et al. on students’ social–emotional skills suggested that social–emotional learning programmes help shape values, strengthen collective belonging, improve interpersonal relationships, promote harm repair, and even impact academic performance (Durlak et al., 2011). Systematic reviews concerning juvenile justice suggest that restorative justice can reduce recidivism among juveniles and gain high recognition from the participants (Robinson & Shapland, 2008), which has positive effects on the prevention and addressing of conflicts and aggressive behaviours such as school violence and school bullying. Supporting evidence can be found in the intervention study by Pérez-Jorge (Pérez-Jorge et al., 2022) and the systematic reviews undertaken by Alonso-Rodríguez (Alonso-Rodríguez et al., 2025).

These reported outcomes are behaviourally plausible because restorative practices can support need satisfaction, emotion regulation, prosocial modelling, and perceived procedural fairness; nevertheless, they should not be interpreted as uniform causal effects across settings.

## 5. Argumentative Synthesis: Adaptation of Restorative Justice in Chinese Schools

International restorative practices suggest that the development of students’ skills in managing healthy relationships and conflict resolution may help them to resolve conflicts before serious incidents can occur. For example, an intervention study indicated that in diverse school contexts, approaches that seek to resolve conflict and repair relationships, rather than merely punish behaviour, have been associated with improvements in coexistence and reductions in violence; their effects remain contingent on context and implementation quality (Avivar-Cáceres et al., 2022). In China, schools rely mainly on a hierarchical accountability system that operates through the enforcement of school rules and the punishment of violations to prevent and respond to student conflicts and injury disputes. It has become a widely adopted practice to expand campus safety supervision by adopting technical means such as AI recognition and cameras. However, as student conflicts such as bullying continue to spread stealthily with low visibility, the egregious nature of some cases has exceeded the bounds of public tolerance. Scholars have gradually come to recognise the inherent physical limitations of hardware-dependent approaches, while policy tools relying on explicit school rules and ex post punitive mechanisms are steadily eroding profound reflection on the essence of education, leaving the pedagogical care that should rightfully be at its core bereft of the respect it deserves. In this context, some scholars have advocated for a paradigm shift in student conflict intervention from an incident-focused to an ecological orientation, constructing a whole-cycle ecological system encompassing prevention, intervention, and restoration (Y. Y. Jiang, 2026), and other scholars have advocated using restorative justice as a means of addressing school bullying (Di & Wang, 2019); however, evaluations of restorative practice in Chinese schools remain scarce.

### 5.1. Limitations of Current Student Conflict Intervention: Taking School Bullying as an Example

Globally, bullying has attracted widespread attention as a typical manifestation of student conflict in school settings (Shen & Wu, 2024). The Chinese government has also adopted multiple measures in recent years to prevent and control school bullying. On the one hand, there is the normative design of legal and policy frameworks. Hardline expressions such as “zero tolerance” and “special campaigns” frequently appear in educational documents and media reports at all levels. Liu’s theoretical work pointed out that laws and regulations such as the Law of the People’s Republic of China on the Protection of Minors and the Regulations on School Protection of Minors emphasise external constraints as a deterrent against individual actions, heighten schools’ educational management responsibilities, and focus on technical correction while neglecting emotional governance (Y. Liu, 2024). On the other hand, there are governance expectations enabled by technological empowerment. The special initiative issued by the Ministry of Education on the prevention and governance of student bullying requires schools to achieve full coverage of video surveillance in concealed areas such as corridors, rooftops and storage rooms (CCTV News, 2024). People have expected technologies such as high-definition cameras, facial recognition, and behavioural analysis algorithms to compensate for the “blind spots” of traditional supervision (Y. Y. Jiang, 2026). However, surveys conducted by scholars in six rural primary and secondary schools in Hunan Province reveal that approximately 63.4% of covert bullying incidents occur in these schools without surveillance coverage (Q. Zhang, 2021). Some scholars have questioned whether such surface-level governance approaches—relying on technical means or the severity of punitive measures—can truly address the deep-seated attributes of conflict intervention.

From the perspective of current educational rule-of-law practices, China’s intervention model for student conflicts predominantly reflects a conceptual orientation prioritising “security first” and “punitive deterrence”. Some scholars frankly point out that at the physical level, access control systems, round-the-clock surveillance, and one-click alarm devices have constructed an impenetrable line of defence; at the institutional level, reporting and disciplinary systems have become expedient instruments for teachers and schools to avoid accountability whenever an incident occurs (Q. Zhang et al., 2024). As Liu notes, technological governance translates the social facts of school bullying into figures, tables, and documents, whereby accountability oversight, digital performance metrics, and standardised governance under bureaucracy constitute key dimensions in school bullying governance practices (Y. Liu, 2024). While this approach helps prevent surface-level student conflicts to a certain extent, it fails to address the relational alienation and rights conflicts underlying them. Moreover, by neglecting these deeper issues, it significantly narrows the educational opportunity to transform conflict incidents into vehicles for life education, empathy cultivation, and enhanced conflict resolution capabilities (X. Wu et al., 2015). For instance, applying uniform punitive procedures to conflicting parties with different psychological states may instead intensify the conflict; school rules designed to address traditional verbal and physical conflicts seem ill equipped to effectively regulate new types of conflicts emerging in the short-video era (such as cyberbullying and violence), and requirements imposing accountability for failure to report incidents may also have the unintended effect of fostering among some educators the belief that “conflict information should not leave the school” or that “only serious incidents warrant reporting”. Theoretically, restorative justice rejects punitive-centred “adversarial justice”, shifting the focus from power-based control to relationship repair. It helps remedy the current tendency in conflict intervention to prioritise surface-level symptoms over substantive root causes, thereby rebuilding student trust and restoring campus order.

### 5.2. The Value and Contextual Opportunities of Restorative Justice in China

First, restorative justice advocates the return of student conflicts to their essence as coexistence and relational problems, which helps to resolve conflicts at the root. In the process of learning, children watch their peers closely, not only for guidance and assurance but also inevitably as a point of comparison. Both work and play are defined as competition and cooperation (Burford et al., 2019). School conflict is an everyday phenomenon in classrooms, but difficulties tend to arise when such conflicts cannot be adequately acknowledged and resolved, or when violence is used. Student injury disputes such as school bullying and violence are often rooted in relational breakdowns linked to imbalances of power and status among students. A fuller understanding of the causes of damaged relationships, together with efforts to repair them, is therefore of considerable importance. In restorative justice, processes and outcomes that promote, develop, and support “relational justice” are sought out, with full consideration given to the background, causes, and circumstances of incidents, which may reveal more complex attributions of responsibility. For example, based solely on surveillance footage, a homeroom teacher may attribute blame to Party B for striking Party A; however, in reality, it was Party A who—harbouring resentment after an unsuccessful attempt to extort money from Party B—deliberately provoked Party B through verbal taunts to induce Party B to strike first (Shi et al., 2025). In an ideal restorative process, Parties A and B fully articulate their perspectives on and needs arising from the incident. To clarify the full picture of events, the teacher or other facilitators guide both parties to understand the harm caused by the conflict, assume corresponding responsibility, and commit to making positive changes.

In this process, relational justice is realised through concrete mechanisms of multilevel relationship repair. First, the reconstruction of horizontal egalitarian relationships. Through respectful dialogue, Party A is expected to relinquish the defensive narrative of “he struck first,” sincerely assuming responsibility, while Party B may resume normal campus life with credible assurances of safety, allowing both parties to begin reconstructing an egalitarian relationship grounded in mutual recognition. Second, vertical institutional trust should be repaired. While guiding the dialogue between both parties, the teacher reflects on institutional oversights—such as why Party A’s pattern of soliciting money went undetected and whether routine pastoral care was adequate—thereby signalling to students that the school aspires to be a just and caring community and helping to rebuild students’ trust in the school system. Third, the reshaping of collective coexistence norms. Social learning theory posits that observational learning in any organism is a dynamic process of reciprocal interaction among the individual, the environment, and behaviour, with individuals regulating their conduct on the basis of behavioural standards conveyed through the social system (Bandura, 1991). Restorative dialogue unfolds with the measured participation of the classroom community, enabling other students to observe that conflicts can be constructively resolved—thereby replacing distorted subcultures of “silent tolerance” or “violence begets violence,” strengthening the class’s norms of coexistence and extending relational justice from the individual to the collective level.

Second, restorative justice meets the practical needs of school dispute resolution in China. Drawing upon the Supreme People’s Court’s representative civil cases involving campus governance and more than 100 school-bullying-related judicial rulings systematically reviewed by the author, it can be observed that current disputes that arise from student conflicts involve multiple parties, and identification, investigation, and litigation processes are time-consuming (Supreme People’s Court of the People’s Republic of China, 2025; X. Y. Chen et al., 2025). Zhang’s qualitative research, grounded in routine cases he encountered while working at a campus dispute mediation institution in Beijing, notes that student injury cases frequently devolve into deadlocks characterised by protracted compensation claims from parents, the evasion of responsibilities by schools, and inefficient mediation by regulatory authorities (X. M. Zhang, 2025). In some cases, the prolonged failure to reach consensus among students, guardians, schools, insurance companies, and medical institutions not only exhausts all parties physically and mentally but also disrupts the harmonious relationships among students and those between schools and students, which affects both study and life. One representative case mediated by a Beijing mediation body involved a student injury incident that lingered unresolved for more than one year, as the forensic appraisal institute could not deliver a definitive evaluation. During multiple rounds of supplementary submission of forensic appraisal materials, the claimant also challenged the professionalism of the medical institution (X. M. Zhang, 2025). In another school bullying case in Hubei Province, the victim developed PTSD and depression. Litigation was eventually filed after compensation negotiations among multiple guardians and the school broke down (Hubei Provincial High People’s Court, 2025).

One empirical study indicated that in some regions, civil disputes are often addressed through informal, intracommunity self-mediation mechanisms (Shi et al., 2025). However, owing to the absence of effective institutional constraints, such self-mediation is frequently accompanied by verbal abuse and physical confrontation (Shi et al., 2025), which extends to student disputes and leads to the escalation of conflicts. Moreover, the harm caused by student conflicts—such as psychological trauma, fractured relationships, and loss of safety—is difficult to quantify in monetary terms. According to the author’s previous empirical research, in judicial practice, the gap between the compensation for emotional damage claimed by parents and the amount awarded by courts remains considerable (X. Y. Chen & Han, 2024); consequently, traditional tort-based damage awards fail to adequately address victims’ practical needs for psychological redress and interpersonal repair (X. Y. Chen & Gao, 2025).

Restorative justice enables the involvement of all relevant parties to discuss the “repair of harm”, with decision-making being carried out by both lay and legal actors (Daly, 2002). This essentially involves constructing actual (rather than symbolic or virtual) communication mechanisms, which create processes in which communication between and among the parties (the victim, offender, their support communities, interest communities, and the broader society) is both possible and probable (Llewellyn & Downie, 2011). A review of the empirical literature on youth conferences finds that conferences reduce victims’ anger and fear toward offenders, more so than courts. For offenders, there is a higher degree of perceived procedural justice and restorative justice in conferences than in court processes (Daly, 2008). It can help address the practical difficulties that often arise in conflict disputes, such as vague fact-finding, divergent compensation standards, and controversial disciplinary processes.

Specifically, drawing on the foregoing theoretical work and logical reasoning of restorative justice, regarding psychological repair, restorative dialogue enables victims to fully recount their experiences and impacts in a safe setting, fulfilling their psychological need “to be taken seriously”. Through listening, offenders gain direct perceptions of the emotional consequences of their actions, triggering remorse and the motivation to repair. The empathic engagement and emotional transformation between the parties, together with the collective identity established among stakeholders through joint dialogue, help break the vicious cycle “silence—isolation—trauma solidification”. With respect to compensation standards, restorative justice transcends the logic of monetary quantification by orienting towards the victim’s genuine reparative needs, whereby the parties involved negotiate a multifaceted reparation plan encompassing apology, behavioural commitment, monetary compensation, and other elements—thereby circumventing the deadlock over compensation benchmarks.

With respect to disciplinary procedures, restorative justice serves as a complement to traditional punitive thinking and accountability models. Traditionally, discipline was thought of as an individual’s ability to adhere to a set of school or classroom rules that were put in place to maintain good order, which is necessary for effective teaching and learning. The traditional approach seeks to ensure accountability through the moral censure that comes with punishment, which assumes that a choice is required between punishment and its lack. However, relying on punishment to convey moral messages is an ineffective form of communication. Restorative justice does not reject punishment but rather holds that individuals have the ability to be thoughtful and considerate concerning their behaviour and how it affects both themselves and others. Listening, learning and responding appropriately are the responsibilities of the community that has been most affected by harmful behaviour (Morrison et al., 2005). In restorative processes, disciplinary action is no longer framed as “punishment imposed by the school on students”, but rather as “how we collectively work out a solution to this problem”. This participatory structure inherently aligns with the requirements of procedural justice. Parents are no longer rights defenders confronting the school, nor are students mere recipients waiting passively for sanctions. Responsibility is based on the participation of individuals whom both sides care about. The inherent nature of procedural justice effectively improves the acceptance of the outcome, thereby achieving accountability with greater substantive significance.

Finally, restorative justice represents an organic integration of law and education that constitutes a strong response to the development of quality-oriented education and rule-of-law education for juveniles. Restorative justice has grown from dissatisfaction with aspects of juvenile and criminal justice, which is typified by punitive and adversarial court processes and has gradually been recognised as a theory of justice rather than a mere alternative practice. Restorative justice is aligned with certain theories of corrective justice and retributive justice while addressing their deficiencies (Llewellyn & Howse, 1999).

The core philosophy of restorative justice is highly aligned with China’s current legal and policy orientations regarding minor protection and education. The newly revised Law on the Prevention of Juvenile Delinquency explicitly emphasises that approaches to minors should be “based on education and protection”, implementing the guiding principles of education, persuasion, and redemption. Similarly, the Family Education Promotion Law requires family, school, and social education to be closely integrated and harmonised to foster a healthy developmental environment for minors. The 2024–2035 Master Plan on Building China into a Leading Country in Education also requires the establishment of a diversified mechanism for resolving campus safety disputes. Such legal and policy instruments provide a favourable policy environment for the legalised settlement of student conflicts and the implementation of restorative justice.

### 5.3. Cultural Resources of Applying Restorative Justice in China’s School Context

With respect to the localization of restorative justice, domestic scholars hold that it is consistent with China’s traditional culture and has a long practical history (S. H. Jiang & Zhang, 2024). Restorative justice not only conforms to ideas such as “freedom from litigation,” “harmony is precious,” and “loyalty and forbearance” but also aligns with the Chinese people’s centuries-long pursuit of the value of “harmony.” In Confucian thought, people’s sense of integrity and shame is both internal and conscious, and it enables individuals (such as offenders) to recognise their mistakes and correct them sincerely (S. Jiang et al., 2007), which coincides with the view of moral internalisation in reintegrative shaming theory. When participants discharge their shame over wrongdoing, they can better distinguish right from wrong and achieve self-restraint.

Simultaneously, the practical methods of restorative justice are deeply compatible with the cultures of “face” (mianzi) and “relationships” (guanxi) in Chinese social interactions. Chinese society operates on a “differential mode of association” structured by interpersonal networks (Fei, 2013). Within the “acquaintance society” of a school campus, a conflict is not merely a violation of rules but a disruption of peer relationships. Traditional punitive models often cause one party to lose face, leading to further deterioration of relationships or even triggering retaliatory psychology. In contrast, restorative practices, particularly restorative conferences, provide a safe and respectful dialogical space for all participants. This allows the offending student to admit their mistakes and assume responsibility while simultaneously “regaining face” through sincere apologies and compensatory behaviours. Consequently, this achieves relational repair and harmonious coexistence, making it much more culturally and psychologically acceptable to teachers and students.

Moreover, in cases where mediation is regarded as a practical form of restorative justice, there are records of the use of moral norms to mediate and resolve disputes during the Qin, Tang, and Ming dynasties as well as the Republican period (Yuan, 2017). “Dismissing litigation (息讼)” was widely adopted to clarify rights and resolve disputes between the state and the people. Dismissing litigation reflects the mainstream Confucian ideology that views lawsuits as morally undesirable. Officials and local elders actively discourage people from bringing cases to court, preferring mediation, compromise, and social harmony over adversarial proceedings. The idea was to “settle grievances without litigation,” preserving community relationships and social order.

The philosophical kinship between 息讼 (dismissing litigation) and restorative justice lies in their shared rejection of adversarial, zero-sum conflict resolution in favour of relational healing. Their key similarities follow: First, they prioritise relationship repair over retribution. Both ideologies view the breakdown of social bonds as the core problem. Restorative justice asks how harm can be repaired; dismissing litigation asks how conflict can be dissolved before it ruptures community harmony. Neither is primarily concerned with determining winners, losers, or proportional punishment. Second, community-centred resolution. Restorative justice brings together offenders, victims, and community members in dialogue. Similarly, dismissing litigation historically relied on elders, mediators, and communal pressure to negotiate settlements outside formal courts. Both reject the state’s monopoly on dispute resolution, treating justice as a collective responsibility rather than a bureaucratic procedure. Third, forward-looking orientation. The idea of dismissing litigation focuses on whether villagers can coexist harmoniously in the same community in the future. Restorative justice centres on three core goals: preventing offenders from reoffending, helping victims rebuild their lives, and restoring a sense of security to the community. The ultimate end of justice lies not in the completion of punishment but in the reconstruction of social relations. Finally, a holistic conception of harm. The principle of dismissing litigation holds that lawsuits rupture interpersonal bonds and corrupt rural social ethics. Similarly, restorative justice emphasises that the emotional trauma, broken trust and collective fear caused by crime are often far more profound than material losses. Both approaches refuse to reduce harm to an abstract legal object defined by legal provisions and instead insist on recognising its social and emotional dimensions.

Moreover, China’s school environment has leadership advantages in terms of the implementation of restorative practices. Leadership has been identified as the single most critical aspect of school reform, and it relates to the formulation and implementation of school norms, as well as value shaping and school culture construction. Influenced by the Confucian traditions of “respect for teachers and education,” head teachers are not only representatives of authority but are also endowed with the moral and educational duty of “transmitting wisdom, imparting knowledge, and resolving doubts.” On the one hand, head teachers possess a natural prestige in the eyes of students and act as symbols of classroom order, making them appropriate facilitators and mediators for restorative justice (Xue, 2007). On the other hand, restorative practices serve as a modern tool for teachers to fulfil their responsibilities of “transmitting wisdom” and “resolving doubts.” Treating conflicts as educational opportunities perfectly aligns with teachers’ professional ethics and role expectations. In daily teaching, head teachers often communicate with students via evaluative language such as “right and wrong,” “good and bad,” and “superior and inferior.” These teachers develop educational life through award evaluations, academic grading, and daily behaviour assessments. This constant state of evaluation has deeply rooted the need for positive feedback in school culture. Thus, students expect clear indications of moral agreement and support as well as appreciation from their head teachers, and thus they follow teachers’ structured arrangements and guidance for restorative practices. Teachers can also use their authority to stabilise the emotions of both parties, play a positive mediating role in the handling of peer conflicts, and encourage students to improve unreasonable behaviours.

In addition, the communication guidance and negotiation mediation strategies that are adopted by teachers in student conflict intervention embody the spirit of restorative justice. In some cases, teachers act as third parties to help conflicting parties communicate frankly and encourage students to propose solutions independently, thereby achieving problem-solving or win-win outcomes (D. L. Zhang et al., 2022). For instance, in physical conflicts such as bullying, some head teachers act voluntarily as conveners and mediators, gathering relevant parties, which include parents and classmates, to understand the causes and consequences and to express their views on right and wrong and on pertinent solutions, a dynamic that resembles the organisational structure of restorative practices. Some authoritative phrases used by teachers—such as “You shouldn’t bully your classmate,” “You should apologize publicly” or “This matter is over. We should be broad-minded” (X. Y. Chen & Han, 2025)—can evoke a sense of shame in wrongdoers, which prompts them to correct their behaviour to a certain extent, which aligns with restorative justice’s focus on harmonious relationships and fostering a school atmosphere characterised by love and empathy.

Such cultural resources should be treated as conditional rather than inherently facilitative: appeals to harmony, face, guanxi, or teacher authority may also pressure students to reconcile, suppress victims’ accounts, or reproduce unequal participation. Several scholars contend that restorative practices may inadvertently mirror and exacerbate existing power dynamics, creating artificial hierarchies that were not previously present (Lyubansky & Shpungin, 2015). In the Chinese context, where “harmony comes first,” victims may face intense pressure from families, school authorities, and community networks to accept apologies and reconcile, lest they be perceived as “disrupting harmony” (Yuan & Aertsen, 2016). The victim’s refusal to reconcile could be interpreted as defiance against collective values, effectively silencing their authentic voice. Meanwhile, the culture of mianzi can also undermine the authenticity of dialogue and accountability. Qualitative studies by several scholars have observed that East Asian individuals’ desire for face-saving becomes especially acute when members of their own culture are present (M. Chen, 2019). In restorative conferences, offenders may offer apologies primarily to preserve or regain face rather than out of genuine remorse, while victims may feel pressured to grant forgiveness publicly to avoid appearing ungracious or vengeful. In school contexts, this means that face-driven apologies may become performative rituals that satisfy the procedural requirements of restorative justice without achieving genuine moral transformation. The victim may experience secondary victimisation when compelled to accept a public apology that feels insincere but cannot be refused without violating social expectations of magnanimity. As several scholars observed when restorative justice was first introduced to China, the Chinese practice of restorative justice shows limitations in aspects such as “coerciveness; lack of full information for offenders; offender’s incompetence; insufficient protection of offenders; and conflicts between victim empowerment and offender rights” (J. Liu & George, 2009). The introduction of restorative justice into Chinese schools may entail similar challenges. Moreover, the authority vested in teachers may also carry the risk of substituting authority-driven compliance for authentic moral reasoning. This point will be elaborated in the subsequent section on “Implementation Challenges in Chinese Schools.”

In sum, the cultural resources of harmony, mianzi, guanxi, and teacher authority provide a fertile ground for restorative justice in Chinese schools, but they are double-edged swords. When cultural values prioritise collective harmony over individual voice and when face-saving motives substitute for authentic remorse, restorative practices may devolve into “control restorative models” (Lo et al., 2005). Any school-based restorative process must protect voluntariness, independent voice, and the right to decline direct dialogue.

## 6. Discussion: Conditions for Safeguarded Implementation of Restorative Justice in Chinese Schools

In education, restorative practices are often touted as a way to improve school climate and academic outcomes while addressing disciplinary issues and equity gaps (Fronius et al., 2019). Although these outcomes are meaningful, they are byproducts rather than the goals of restorative practices (Mustian et al., 2022). Presently, some schools in China are attempting to implement restorative practices, and some courts are also using restorative strategies to resolve student disputes. The application of restorative justice in schools is in a stage of exploration and development. The implementation of restorative justice in student conflict intervention requires addressing the challenges derived from traditional school culture and should depict practical models of restorative justice through the combination of the reality of China’s schools and international experience.

### 6.1. Implementation Challenges in Chinese Schools

Intervention evaluations have produced mixed evidence. A pilot randomised trial indicates that restorative justice decreases the frequency of violent incidents by providing a safe space for conflict resolution (Bonell et al., 2014, 2015). A two-year cluster-randomised trial of restorative practices found no significant differences between schools that implemented restorative practice and those that followed traditional disciplinary methods (Acosta et al., 2019). This discrepancy suggests that the success of restorative justice may depend on contextual and implementation factors, such as teacher commitment, programme structure, and underlying school culture.

From a behavioural science perspective, this variability is unsurprising: sustainable change requires students to experience autonomy, competence, relatedness, and fair treatment while observing non-violent, accountable ways of resolving harm. A programme that adds a conference to an otherwise punitive environment is therefore unlikely to alter learned expectations or norms.

Some overseas practices also indicate that the key to implementing restorative justice lies in achieving cultural change within schools and shifting away from the mindset associated with traditional discipline towards that associated with restorative discipline (Morrison et al., 2005) to transition from the use of punitive or reward-based external motivators to that of relational motivators. This shift poses a major challenge to schools in China, which have long been deeply rooted in the Confucian ethical tradition of “the dignity of teachers” and external incentive mechanisms.

First, rooted in the value tradition of teachers’ dignity, schools have tended to develop authoritative interpersonal dynamics between teachers and students as well as a paternalistic approach to management and discipline. The Book of Rites (Liji) states, “Only when the teacher is stern is the Way honoured; only when the Way is honoured do the people know to respect learning.” This has traditionally been understood as binding the teacher’s authoritative status to the transmission of the “Way” (dao), granting the teacher moral authority beyond that of a mere transmitter of knowledge. Within this tradition, the teacher is positioned as a transmitter of culture who “speaks on behalf of the sages”, while the student is expected to abide by the hierarchical order with an attitude of “serving the teacher as one would serve one’s father”. In effect, this ethical configuration tends to cast the teacher–student relationship as a quasi-kinship hierarchical sequence, in which the teacher’s authority is recognised not only as a functional necessity (effective teaching) but also as a moral obligation (cultural transmission).

Since modern times, with the scaling of school organisations, the teacher has increasingly been embedded within the administrative chain of “principal—homeroom teacher—subject teacher”, coming to serve as the specific implementer of school rules and regulations and the primary bearer of responsibility for classroom order. The teacher thus tends to assume the dual roles of disciplinarian and adjudicator and acquires dual authority grounded in both knowledge and discipline. The teacher typically holds the power to interpret rules, evaluate conduct, and decide upon rewards and punishments, while the student occupies a relatively passive position of “being evaluated”.

While teachers’ authority helps them act as qualified mediators, it also makes Chinese teachers accustomed to using cessation strategies rather than mediation strategies (such as restorative justice) in handling peer conflicts. Cessation strategies are interventions that focus on the external management of conflict situations, specifically ending disputes by telling or directing children what to do or by physically separating them (D. L. Zhang et al., 2022). Teachers who apply cessation strategies often give firm instructions, such as “Apologize to him,” “Go write a self-criticism,” or “Ask your parents to come this afternoon.” Such approaches easily overlook the background factors that lead to such conflicts and may cause some conflicts to be misjudged. For example, in the middle school that the author attended, playing basketball in the hallway and arguing with a teacher were interpreted as disobedience or rebellion. If restorative dialogue is conducted with the offending parties, the root cause of the problem (e.g., a lack of social skills, boredom, not knowing the game rules, etc.) can be identified so that the issue can be solved without resorting to punishment. A qualitative study revealed that head teachers in China are already juggling multiple responsibilities, working long hours and facing great pressure, and this results in insufficient time and energy for focusing on conflict prevention and intervention (Q. Zhang et al., 2024). When facing conflicts such as bullying and violence, teachers lack systematic mediation skills and are often caught between the parents of each party.

Second, the educational management model of schools tends to exhibit pronounced externally driven incentive characteristics. In terms of disciplinary management, explicit school rules and ex post punitive mechanisms have largely formed the core policy toolkit, with legislators expecting to reduce student misconduct through the deterrent effect of educational sanctions (Cheng, 2021). In terms of teaching evaluation, quantitative assessment and ranking-based competition serve as the core mechanisms, with external symbols such as scores, rewards and punishments, and honours functioning as the principal instruments of behavioural regulation. Some schools attempt to use observable and quantifiable external behavioural data (e.g., incident rates and the frequency of disciplinary sanctions) to define inherently incommensurable internal emotional experiences (e.g., the severity of trauma and relationship repair) (Y. Y. Jiang, 2026). Although this externally incentive-driven model demonstrates that it can be efficient in short-term behavioural management, it fails to foster students’ autonomous self-discipline—compliant behaviour may rapidly disintegrate once external monitoring ceases or the incentive structure changes.

The external incentive model has led some schools to grow accustomed to pursuing short-term governance outcomes while neglecting the development of campus culture and the cultivation of a positive school climate. It remains questionable whether schools will commit resources to restorative practices and fostering a positive campus culture. Theoretically, school atmosphere refers to the “norms, values, and expectations that support people feeling socially, emotionally, and physically safe” (National School Climate Council, 2007). The atmosphere of a school has been identified as an important factor in the promotion of positive behavioural and academic outcomes for young people and the reduction in negative risk factors (Thapa et al., 2013). The goal of restorative practices is to create a school environment in which individuals take responsibility, understand, support and learn from each other. In this process, the cohesion of the teaching staff and the involvement of the management team are necessary (Duque & Teixido, 2016). School administrators need to embrace restorative campus culture and include restorative practices in their coexistence policies to achieve integration at the cultural level. The first step is to build a consensus, including agreement regarding the following questions: Is conflict an individual or collective issue? Who is responsible for such conflict? How can it be repaired? Shifting perceptions and responses to problems poses significant challenges for schools that tend to quickly quell conflicts. In practice, under the long-standing influence of “exam-oriented education” and the “score-only mentality”, most schools focus primarily on improving student performance and increasing graduation rates rather than preventing student conflicts (Z. J. Liu & Xu, 2020). School resource allocation and curriculum design still prioritise academic performance, with subjects such as Chinese, mathematics, and English taking precedence. In contrast, legal and emotional education—mainly delivered through “Morality and Rule of Law” courses—is often regarded as insignificant or even dispensable (Li, 2020). Unlike academic achievements, conflict prevention is an “invisible” task with no immediate, measurable “performance” outcomes. Officially issued by the State Council in June 2026, the Outline Plan for Education Development During the 15th Five-Year Plan Period proposes to “further advance well-rounded education” and “abandon the exam-oriented view of educational performance and foster a sound educational ecosystem”. In line with the plan’s requirements, schools will gradually prioritise the cultivation of students’ social and emotional competence, yet such a transition will take time.

Third, the traditional mindset and rights-defending attitudes of parents constitute another practical obstacle to the implementation of restorative practices. Existing research indicates that families are another crucial aspect of maximising the impact of restorative practice (Weber et al., 2021). However, how to achieve effective parental participation rather than mere blame constitutes a key consideration in the design of restorative procedures. Influenced by protective psychology and the naive sense of justice akin to “an eye for an eye,” parents’ primary demand when their children experience conflict is often to “seek an explanation,” pursuing clear accountability and punitive compensation (Fu et al., 2026). Some parents even label ordinary playful scuffles among students as bullying incidents and pressure schools and teachers to expel the so-called “bullies” (Q. Zhang et al., 2024).

This adversarial approach to defending their rights can make it difficult for them to understand and accept the dialogue, empathy, and relational repair advocated by restorative justice. They may interpret the restorative process as an ambiguous “smoothing over of differences” or an “unfair” practice that blames both sides equally. In their view, such an approach not only fails to effectively punish the offender but also may inflict secondary trauma on the victimised child by failing to adequately validate their grievances. In such cases, some parents exert pressure on schools, demanding the adoption of the “hard measures” they endorse—such as public apologies, disciplinary records, and financial compensation—sometimes escalating student conflicts into confrontations between families and schools. This pressure may lead schools and teachers to shift much of their focus towards pacifying parents’ emotions and mitigating risks rather than emphasising the educational value of the conflict and the long-term development of the students.

### 6.2. Implementation Suggestions in Chinese Schools: Prevention and Intervention Programmes

Given the development stage and challenges posed by restorative justice in China, it is difficult to fully implement restorative practices in schools. A step-by-step approach involves gradually building a restorative school culture through curriculum design and typified interventions. To overcome the initial resistance of students and teachers, who may be accustomed to traditional disciplinary approaches, sensitization and training should be implemented before restorative practices are introduced to ensure consensus among all community members in understanding and accepting the benefits of this approach.

**Figure 1:**
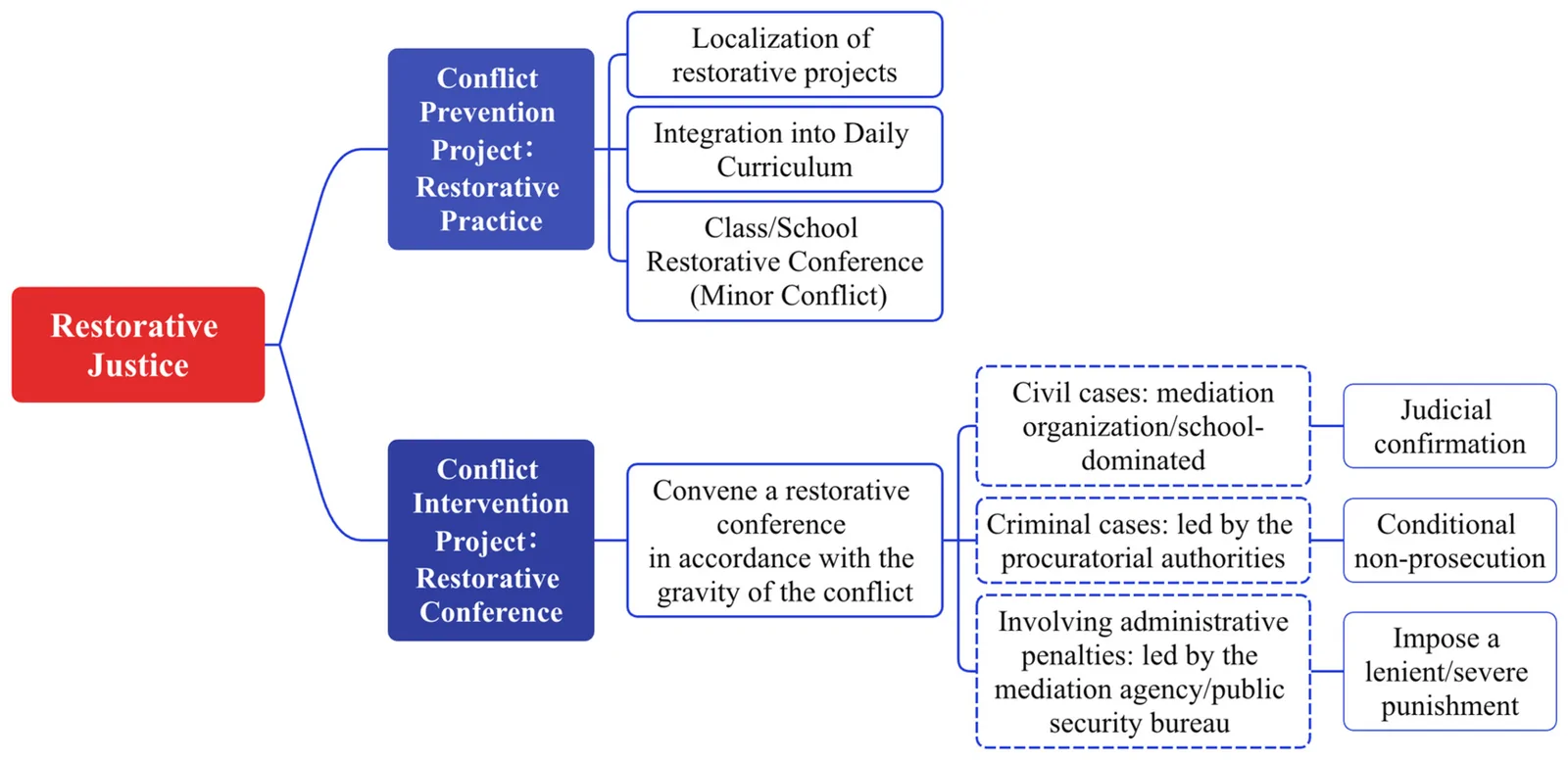
Restorative Justice in Chinese Campuses.

In school settings, the implementation of restorative practices is not simply a case of overlaying a justice model based on conferencing or one-off interventions. Rather, it should be a continuous practice in which restorative education is gradually integrated into school norms and curriculum systems to consolidate its positive impact on school climate. Schools are not only a microcosm of society but are also independent face-to-face communities composed of complex interpersonal relationships. Unlike the criminal justice setting, where victims and offenders may not know each other or see each other again, in schools, the victims and offenders will most likely see each other the next day. In the context of these tightly meshed communities, harmful behaviour not only has implications for the individuals directly involved but also has far more direct implications for the wider web of relationships that exist within schools. If not comprehensively addressed, small conflicts may escalate rapidly. Therefore, restorative practices must be gradually integrated into school culture to form a sustainable practice system. Specifically, in reference to common international practices, restorative justice in schools can be separately implemented as prevention and intervention programmes. Figure 1 below illustrates the operational framework of restorative justice on Chinese campuses conceived by the authors.

(i) Prevention programmes. Prevention programmes focus on the development of prosocial behaviours for all students and aim at improving students’ skills in conflict resolution and social–emotional development, thereby cultivating the “conflict intelligence” required to effectively handle low-to-medium level disruptive, complex, and persistent conflicts (Y. Wu et al., 2025). Prevention programmes permeate the entire process of education and teaching and can broadly be implemented in three ways. The first approach is to roll out a series of restorative justice programmes. Under the overall leadership of provincial education authorities, schools may appropriately introduce internationally mature restorative programmes for curriculum design and norm adjustment, such as Morrison’s Responsible Citizenship Programmeme (RCP) and the Olweus Bullying Prevention Program (OBPP), social and emotional learning program (SEL), and so on. The content focuses on in-depth analysis of child and adolescent developmental psychology, scenario-based practical exercises in restorative justice conferencing, core concepts and school-based applications of trauma-informed education, and psychological first aid and long-term supportive counselling for students following crises, with an emphasis on cultivating professionals’ ability to gain insight into students’ psychological needs, resolve deep-seated interpersonal conflicts, and repair psychological trauma.

The second approach consists of integrating restorative practices into daily curricula. During curriculum development, these concepts should be subtly integrated into the local educational context to develop teaching tools that fit the Chinese cultural environment. For instance, training in emotion management, nonviolent communication, and empathy can be systematically introduced in class meetings and Morality and Rule of Law classes. In daily interactions, teachers should frequently use “restorative questions”—such as “What were you thinking at the time?” or “How do you think this affected others?”—to guide students in shifting from a blame-oriented mindset of “who is right and who is wrong” toward a reflective mindset focused on “impact and responsibility,” thereby creating a campus language culture that encourages expression and reflection.

These universal activities should be explicitly designed to support autonomy, competence, and relatedness and to make constructive conflict resolution observable and reinforced in daily peer interactions. This connects curriculum delivery to the mechanisms specified by self-determination and social learning theories.

The third approach is to appropriately employ restorative justice conferences to address student conflict incidents on campus. In conflict skill training, special attention should be given to the role and value of peer mediation. On the basis of appropriate teacher guidance, students’ autonomy and decision-making power should be fully respected to cultivate their independent and responsible decision-making ability. For example, to address minor friction and verbal altercations, more senior students or student leaders can be trained as mediators to rapidly resolve conflicts within the class using informal restorative dialogues, thus preventing the escalation of incidents. Because restorative practices require participants to possess certain capacities for empathy, verbal expression, and reflection, when such programsprograms are implemented, the focus should be placed on cultivating students’ emotional expression, concepts of justice, understanding of restoration, conflict resolution and mediation skills, and restorative justice role-playing (Morrison, 2003).

(ii) Conflict intervention programmes. Conflict intervention programmes target continuous or multiparty complex disputes, such as student conflicts that involve bullying, violence, assault, and isolation that have already caused damage. To address these issues, schools may form a “Restorative Practice Support Group” consisting of systematically trained professionals, such as school psychologists, moral education directors, and vice-principals for legal affairs, to act as a neutral third party that leads and escorts the restorative process. With respect to participant composition, this group actively invites parents to attend conferences and supplements this with regular parent workshops. By explaining the philosophy and value of restorative justice and clarifying how it focuses on children’s growth rather than mere punishment, the group proactively transforms this key group of stakeholders into allies in the restorative process (Y. Y. Jiang, 2026).

Before any restorative dialogue is considered, the support group should conduct and document a case-specific safeguarding assessment. Participation must be voluntary and based on age-appropriate informed consent; no student should be pressured to meet, apologize, forgive, or disclose information. Victims should be able to choose indirect participation, a support person, or no participation, and the limits of confidentiality, including mandatory reporting, must be explained in advance. Cases involving immediate safety risks, serious violence, repeated bullying and other high-risk scenarios should first be referred to the corresponding child protection, disciplinary or legal procedures. A restorative process may be considered only when safety is secured and when it cannot compromise the student’s rights or the school’s legal duties. Specific exclusion conditions include: (a) cases involving serious harmful incidents, sexual assault, or repeated predatory bullying, where the victim faces ongoing physical or psychological danger; (b) situations characterised by coercive relationships or marked power asymmetries that cannot be adequately neutralised through facilitator intervention, such as cases where the offender holds significant social or institutional leverage over the victim; (c) incidents requiring mandatory reporting under child protection laws, where the school’s legal obligation to report to authorities takes precedence over internal resolution; and (d) cases where the offender demonstrates an egregious attitude, refuses to acknowledge wrongdoing, or the victim explicitly declines participation (Wolthuis et al., 2020).

In terms of constructing the dialogue mechanism, the support group can select restorative conferences such as victim–offender dialogue, family group conferences, and restorative justice circles in accordance with the nature and severity of cases (Specific case types and applicable procedures for restorative justice are detailed in Section 6.3), which help those involved in a conflict understand that conflict is more constructive. Through the participation of a third party (mediator), relevant stakeholders are encouraged to sincerely express their accounts of the incident, their respective feelings, and the impacts they have endured. Collaborative alternatives are sought to solve student conflicts so that all the parties share the responsibility for achieving relationship repair and peaceful coexistence. The aim is to develop safe school communities that are more responsive and more restorative. When restorative conferences are held, guided dialogue patterns and driving strategies are used to encourage sincere expression and create an environment that is conducive to effective communication (X. Y. Chen & Han, 2025). Furthermore, the basic requirements of restorative justice, such as information transparency, truth-telling, empowerment, and vindication, should be met as much as possible (Zehr, 2002). Factors affecting fairness (or procedural justice), such as both parties being treated fairly, being treated with respect, and being listened to, should also be valued (Daly, 2008).

### 6.3. Implementation Suggestions in Chinese Schools: Essential Conditions and Considerations

First, the limits of restorative justice in school settings should be clearly specified. According to China’s educational and legal systems, student conflicts can be categorised into the following types according to their severity. (a) Minor conflicts, which refer mainly to conflicts that have not yet violated the Public Security Administration Punishments Law of the People’s Republic of China and the Criminal Law of the People’s Republic of China but have violated normative documents such as the Code for Primary and Secondary School Students and the Code of Daily Conduct for Primary and Secondary Schools or school discipline. (b) Moderate conflicts, which refer to behaviours that have violated the Public Security Administration Punishments Law of the People’s Republic of China but have not yet breached the Criminal Law of the People’s Republic of China or acts that have caused physical or mental harm that constitutes civil infringement. (c) Serious harmful incidents, which refer to behaviours that violate the Criminal Law of the People’s Republic of China, such as conflicts that are suspected to be crimes of intentional injury, insult and defamation, or robbery (X. Y. Chen & Han, 2026). The three categories of conflicts and their representative behaviours are presented in Table 1 below.

Minor conflicts are most suitable for restorative practice intervention. Informal approaches such as restorative questioning, peer mediation, and class-meeting education can be used to prevent escalation. The traditional “victim–offender mediation model” can be applied to this type of behaviour, limiting the scope to the class teacher or head teacher, the two parties involved in conflict and their parents to minimise the impact of conflict and reach an agreement as quickly as possible. If both parties reach a restorative agreement, the class teacher shall oversee and assist with its implementation. Examples include creating an appropriate setting for a public apology during class meetings, observing behavioural changes among the parties to the conflict, and encouraging the isolating student to proactively invite the targeted peer to participate in group activities. Moderate conflicts and serious harmful incidents require formal restorative procedures (e.g., restorative conferences) supplemented by safeguarding assessments, parental involvement, and school records. For these conflicts, the severity of violence and the consequences of legal damage are greater (Y. H. Chen & Xu, 2022), with a serious and wide impact, so that communication alone cannot solve the problem completely; thus, the “family/roundtable meeting model” can be applied. Crucially, the initiation of restorative procedures for such cases must comply with the safeguarding assessment requirements and restrictive conditions outlined above. The judicial authorities will preside over the proceedings, and schools, teachers, anti-bullying expert communities and other parties will be included in the meeting (X. Y. Chen & Han, 2025). It should be noted that for serious harmful incidents, legal procedures should take priority in principle. The police should be notified immediately, and mandatory reporting procedures must be initiated. Restorative conferences may only serve as a supplementary procedure within the criminal or administrative process, and their use shall be determined by public security and judicial organs in light of the opinions of both conflicting parties.

Second, effective linkage mechanisms between restorative conferences and judicial proceedings should be established. The concern that restorative approaches are too soft also stems from the fact that this might let the individuals who are largely responsible off lightly, or perhaps even free (Llewellyn & Downie, 2011). Even if they are still seen as partly responsible, critics might argue, the ability to shine the light of responsibility elsewhere will reduce accountability for those individuals most responsible. The restorative justice approach aims to ensure responsibility and accountability, so that harms stemming from wrongful conduct can be recognised and addressed. Restorative outcomes must be translated into structured, tiered, and monitored agreements rather than symbolic apologies. To safeguard against superficial compromise, clear linkages between restorative conferences and formal accountability structures should be established; see the lower section of Figure 1.

For instance, in civil cases (Category b cases), mediation organisations for campus disputes may facilitate the conclusion of restorative agreements, with the compensation portions being subject to judicial confirmation, thereby lending enforceability to the relational outcomes of dialogue. In incidents that may trigger the application of the Public Security Administration Punishments Law (Category b cases), if the offender sincerely repents, actively assumes responsibility, makes positive reparations to the victim, and obtains forgiveness during the restorative conference, the administrative authority may exercise discretion within the statutory range to mitigate or reduce penalties. It should be stressed that the decision to participate in a restorative conference must remain genuinely voluntary. Saulnier and Sivasubramaniam’s empirical study demonstrates that coerced participation undermines the authenticity of dialogue and the sincerity of remorse: offenders who feel compelled to apologise exhibit lower levels of personal responsibility and accountability, while victims pressured to forgive report diminished perceptions of procedural fairness (Saulnier & Sivasubramaniam, 2015). Consequently, a refusal to participate in restorative conferencing—whether by the offender or the victim—should not in itself trigger harsher disciplinary or legal sanctions. The legitimate basis for aggravation lies in the offender’s conduct and attitude during the process: if, having voluntarily participated in the conference, the offender displays an egregious attitude, publicly insults the victim, or repudiates a duly reached agreement, the public security bureau may consider imposing an appropriately heavier penalty within the scope of its discretion, thereby upholding the educational and deterrent functions of the law. In criminal cases (Category c cases), restorative conferences and agreements led by procuratorial organisations may serve as reference factors for the conditional non-prosecution of juvenile offenders. This approach is already practiced in jurisdictions such as New Zealand, where family group conferencing occurs within a legal framework that retains state oversight (European Forum for Restorative Justice, 2022). Here, too, the principle of voluntariness is paramount: conditional non-prosecution should be available only to those who voluntarily engage and demonstrate genuine accountability through the process, not withheld as a punitive threat against those who decline. This bidirectional linkage—under which restorative engagement may lead to mitigation but non-engagement merely resumes standard legal procedure without additional penalty—ensures that legal obligations remain in force while their application is dynamically informed by relational outcomes. This linkage confirms that restorative justice and proportionate accountability are not mutually exclusive; instead, they are dynamically interdependent within the responsive regulation pyramid (Braithwaite, 2002). Meanwhile, it is necessary to clarify that a restorative justice process should be conceived of as more than a one-off conference or meeting because relationships are fluid, dynamic, and ever-changing. It will involve preparation and follow-up work but also, depending on the situation and the relationships affected, it may involve more than one encounter in a given process. One might have to bring the same parties back together more than once to arrive at an acceptable outcome plan (Llewellyn & Downie, 2011).

Third, the training and capacity-building of restorative justice should be stipulated. In accordance with restorative practice guidelines issued by WestEd and other organisations, effective restorative-practice implementation rests on a four-tier capacity-building framework that moves beyond one-time technical training to sustained professional development. (i) Adaptive training addresses paradigm shifts—challenging punitive, individualistic mindsets and cultivating a relational ethos of shared power and collective responsibility (Trout, 2021). It requires educators to critically examine their own beliefs about student behaviour and to recognise that resistance to restorative practices often stems from a deep-seated attachment to control-oriented disciplinary logic. Schools must therefore create reflective spaces where staff can interrogate how traditional power structures have shaped their interactions with students and commit to embodying restorative values as a daily way of being rather than treating them as a programmatic add-on. (ii) Relational training builds the social capital required for authentic dialogue, including skills in trust-building, deep listening, and vulnerability (Harnetiaux et al., 2024). By normalising vulnerability and establishing agreements that uphold individual dignity, participants are better equipped to navigate complexity and difference, lowering the risk of falling into either punitive blame or superficial harmony. (iii) Structural training equips school leaders to reallocate time and redesign disciplinary policies. Schools should create dedicated advisory periods for relationship-building and invest opening weeks in norm-setting rather than premature curricular coverage—an approach that reduces behavioural-management costs over the long term (Trout, 2021; Arets et al., 2016). (iv) Technical training imparts concrete facilitation skills—circle-keeping, affective statements, restorative questioning, and peer mediation—that educators can deploy in classrooms and beyond (Atlantic Philanthropies et al., 2014). Mastery of these techniques includes learning to sequence restorative questions that shift students from defensiveness to reflection. To prevent educator burnout and resistance, schools should also provide tangible recognition—whether financial compensation, dedicated planning time, or leadership pathways—for staff who commit to this intensive professional growth (Trout, 2021).

## 7. Conclusions

This article argues that restorative justice is a promising but conditional supplementary framework for student conflict intervention in Chinese schools. By linking relational justice with behavioural accounts of need frustration, social learning, and emotion regulation, the article reframes student conflict as a relational and developmental issue rather than solely a breach of rules. International studies provide comparative references for possible benefits and implementation limits, but the effectiveness in Chinese schools remains to be investigated in subsequent empirical studies. Its potential local relevance lies in the convergence between its relationship-centred orientation and policy commitments to participation, mediation, education, and diversified dispute resolution, together with the continuing relationships characteristic of school communities. These institutional and relational conditions may support relational repair and social–emotional learning. However, cultural norms associated with harmony, “face”, “guanxi”, and teacher authority may also create pressure to reconcile or produce unequal participation. As some studies have noted, restorative justice is not a panacea, a substitute for the legal system or a solution to all problems (Wang, 2005). Restorative justice should act as a complement rather than a full replacement for the traditional punitive disciplinary model in student conflict intervention. The key to implementing restorative justice in schools is to cultivate a restorative school culture and encourage school administrators and teachers to embrace restorative practices. It must also be emphasised that trained facilitation, risk assessment, voluntary and informed participation, follow-up mechanisms, and formal legal referral and linkage mechanisms are essential conditions for responsible implementation. Schools can draw on restorative prevention programmes to help build students’ conflict resolution skills and on restorative justice procedures to respond to student conflicts. This article is conceptual and normative, not a systematic review or empirical study. Its conclusions are theoretical and practical propositions; the feasibility, safety, implementation fidelity, and outcomes of restorative approaches in Chinese schools require further local empirical evaluation.

## Figures and Tables

**Table 1:** Categories of Student Conflicts.

Categories	Typical Behaviour				
Minor Conflicts	Classroom quarrels and verbal defiance	Name-calling and mild mockery	Small-group exclusion and social isolation	Minor pushing and horseplay Minor offences in online chat groups	Friction arising from disciplinary violations
Moderate Conflicts	Physical altercations causing minor injury	Open insult and defamation	Extortion of money and “protection fees”	Cyberbullying and doxxing Persistent stalking and harassment	Malicious destruction of personal property
Serious Harmful Incidents	Intentional assault causing light injury or above	Robbery and extortion Group affray	Forced humiliation and indecent assault	Picking quarrels and provoking trouble	Insult and defamation of grave severity

## Data Availability

No new data were created or analysed in this conceptual and normative article. Data sharing is not applicable.
